# The Impact of Quality of Life on Oral Health in a Male Prison Population in Portugal: A Cross-Sectional Study

**DOI:** 10.1055/s-0045-1813032

**Published:** 2026-01-20

**Authors:** Diana Meireles, Paulo Rompante, Rosana Costa, Filomena Salazar, Marco Infante da Câmara, Maria Gonçalves, Marta Relvas

**Affiliations:** 1 Department of Medicine and Oral Surgery, University Institute of Health Sciences (IUCS-CESPU), Gandra, Portugal; 2 Oral Pathology and Rehabilitation Research Unit (UNIPRO), University Institute of Health Sciences (IUCS-CESPU), Gandra, Portugal; 3 Associate Laboratory i4HB—Institute for Health and Bioeconomy, University Institute of Health Sciences—CESPU, Gandra, Portugal; 4 UCIBIO—Applied Molecular Biosciences Unit, Translational Toxicology Research Laboratory, University Institute of Health Sciences (1H-TOXRUN, IUCS—CESPU), Gandra, Portugal

**Keywords:** oral health, quality of life, health status, adults, prisoners

## Abstract

**Objective:**

Oral health problems can have a significant impact on people's quality of life, negatively affecting the most disadvantaged populations. The prison population compared with the general population has a higher risk of developing oral health problems. The main objective of this study was to assess the relationship between prisoners' oral health status and their oral health-related quality of life (OHRQoL).

**Materials and Methods:**

This cross-sectional study was conducted with the participation of 103 male prisoners aged between 18 and 70 years. Participants underwent an intraoral clinical examination and responded to the Oral Health Impact Profile (OHIP)-14 and sociodemographic questionnaires.

**Results:**

The sample mean age was 42.7 ± 9.6 years. The impacts of oral health on quality of life were assessed using the OHIP-14, which presented a mean score of 13.16 ± 11.79. The highest prevalence of impact on oral health was observed in the domains of psychological discomfort (29.6%), followed by physical pain (24.3%). It was observed that 68.0% of the individuals had caries lesions. The mean Decayed, Missing, and Filled Permanent Teeth Index (DMFT) was 16.88 ± 8.56, and the component with the highest weight was the number of missing teeth, with a mean value of 13.00 ± 8.44.

**Conclusion:**

In terms of assessing oral health-related quality of life (OHRQoL), it was observed that the higher the value of the different periodontal indices, as well as the number of missing teeth, the worse the quality of life in relation to oral health. Therefore, it is necessary to create measures to promote oral health and self-care.

## Introduction

Oral health is not only a basic right to which everyone should have access, but also a fundamental component for ensuring overall health and well-being, contributing significantly to an excellent quality of life. 1 According to the World Health Organization (WHO), quality of life is defined as “an individual's perception of their position in life in the context of the culture and value systems in which they live and in relation to their goals, expectations, standards, and concerns.” 2 This concept is inherently complex, varying according to each person's perception and shaped by physical, psychological, and social factors, regardless of age. 2 3

Considering these aspects, oral health-related quality of life (OHRQoL) is an important parameter both in the evaluation of the impact of oral diseases on the population as well as interventions by health professionals to improve oral health. 4 Oral health problems can have a negative impact on people's daily lives, and it is clear that they will end up disproportionately affecting the most disadvantaged in society, which can lead to problems with eating, speech, self-confidence, and even social integration. 5 6

In recent years, the prison population worldwide has increased considerably, prompting the need for policy measures aimed at improving the quality of dental services in prisons and ensuring access to good oral health. 6 In many prisons, the conditions of prisoners are not the most favorable; there are many problems in terms of overcrowding, lack of physical exercise, inadequate nutrition, violence or even staying in physical spaces without access to drinking water or electricity. 7 These conditions cause the development of chronic pathologies and the spread of infectious diseases. 8

Compared with the general population, inmates have a higher prevalence of oral diseases, tooth decay, and missing or filled teeth, requiring more treatment conditions. 9 10 Some of the typical behaviors observed, such as excessive smoking, poor oral hygiene, the use of legal or illegal drugs, or incorrect eating habits, may also explain the low rates of oral health. 11 12

Assessment of oral health should not be restricted exclusively to clinical criteria, since quality of life is a meaningful parameter for capturing the broader consequences of oral conditions and guiding effective interventions. 13

While previous studies document a substantial burden of untreated dental caries and periodontal disease among inmates, 11 12 13 14 few have specifically explored the relationship between oral health status and OHRQoL in European prison populations. Thus, critical gaps remain in understanding the multifactorial determinants of OHRQoL within this setting.

The aim of this study is to evaluate the impact of oral health conditions on the quality of life of inmates, using validated measurement instruments such as the Oral Health Impact Profile (OHIP)-14. By focusing on a prison population, this research seeks to identify the key determinants of oral health-related quality of life and to assess specific needs that are often overlooked in traditional care settings.

## Materials and Methods

### Study Design

The present study was conducted on inmates at Paços de Ferreira prison (EPPF) during the period from October 2023 to June 2024. The study was submitted to and approved by the Ethics Commission of the University Institute of Health Sciences, under reference CE/IUCS/CESPU-29/22, and by the General Directorate of Reintegration and Prison Services. Patients were carefully informed through oral and written explanations about the purpose and procedures of the study. Patients who agreed to participate in the study were asked to sign an informed consent and to complete a questionnaire before the clinical examination. The data obtained were identified only by a code, and no one besides the researcher (M.R.) is aware of the person corresponding to the questionnaire.

The following inclusion criteria were considered: male inmates of the EPPF during the data collection period and aged between 18 and 70 years. For exclusion, the following criteria were considered: inmates who suffered from severe mental illness, particularly, schizophrenic psychosis.

The sample size was selected according to a non-probability convenience sampling method from the inmate population of the EPPF. The study population in this cross-sectional observational study comprised 103 male prisoners. The study followed STROBE guidelines. 15

### Data Collection

In this study, information was collected on sociodemographic characteristics such as gender, age, education, marital status, professional activity, criminal record, sentence length, number of visits per month, work in the EPPF, sharing of sleeping quarters, general health behaviors (daily smoking, systemic diseases, medication), and oral hygiene habits and oral health.

### OHIP-14 Questionnaire

Participants were invited to fill in an OHIP-14, validated from Portugal by Afonso et al, 16 divided into seven dimensions: physical limitation, physical pain, psychological discomfort, physical disability, psychological disability, social disability, and disadvantage, with 14 items to determine the quality of life. Answers to this questionnaire were classified on a scale of 0 to 5, distributed as never (0), rarely (1), sometimes (2), often (3), and very often (4). The prevalence of OHIP was assessed considering the number and percentage of study participants who reported having an impact on their OHRQoL by mentioning “frequently” or “very frequently.” The study also assessed the severity and extent of the OHIP-14.

### Dental Assessment and Diagnosis of Periodontal Diseases

During the intraoral clinical examination, the teeth were assessed according to the Decayed, Missing, and Filled Permanent Teeth Index (DMFT Index), considering the diagnostic criteria defined by the WHO. 2 A tooth is classified as healthy when there is no evidence of a carious lesion, with or without treatment.

To diagnose cases of gingivitis and periodontitis, the new American Academy of Periodontology/European Federation of Periodontology (APP/EFP) consensus was used. 17 According to this classification, gingivitis was diagnosed when the bleeding rate reached percentages of ≥10% and a PD ≤3 mm. On the other hand, periodontitis was diagnosed when there was interproximal CAL ≥2 mm on two or more non-adjacent teeth or interproximal CAL ≥ 3 mm on the buccal or lingual/palatine teeth.

Two expertly trained periodontologists (D.M./M.R.) then carry out a periodontal examination. To confirm inter- and intra-examiner reliability, periodontal parameter measurements were repeated on 20% of the sample and compared with those recorded by a gold standard examiner. The *k* coefficients (within 1 mm) between examiners ranged from 0.61 to 0.80 when assessing probing depth (PD) and from 0.81 to 0.99 when assessing gingival recession (REC). Intra-examiner consistency rates for repeated measurements were 0.89 to 0.94 for PD and 0.81 to 0.94 for REC.

The variables collected through the clinical examination were the number of tooth decay, missing and filled teeth, the number of teeth with mobility, presence of PD, gingival recession (REC), clinical attachment loss (CAL), bleeding Index (BoP; Ainamo and Bay 18 ) and Plaque Index (PI; O'Leary et al 19 ). These data were recorded at six locations for each tooth. The recording of third molars was not included in this study.

### Statical Analysis

The data were analyzed using IBM SPSS Statistics Software (Statistical Program for Social Sciences), version 29.0 for Windows.

Descriptive statistics were expressed as mean, standard deviation, median, first quartile, and third quartile for quantitative variables and as frequencies and percentages for qualitative variables. The OHIP responses were dichotomized to calculate OHIP prevalence, which is the frequency of inmates reporting impacts on OHRQoL. Presence of impact was defined as one or more responses reported “often” or “very often,” while responses “never,” “rarely,” and “sometimes” represented the absence of impact.

The normality of the variables under study according to the groups was analyzed using the Shapiro–Wilk test, and their non-normality led to the adoption of a non-parametric analysis.

The Mann–Whitney test was used to compare OHRQoL according to smoking habits and caries lesions. The Kruskal–Wallis test was used to compare OHRQoL according to age group, number of cigarettes per day, oral hygiene habits, perception of oral health, and periodontal status, followed by the Dunn test with Bonferroni correction. The Spearman correlation coefficient was used to assess the relationship between OHRQoL, periodontal indices, and the number of missing teeth. The level of statistical significance used was *α* = 0.05.

## Results

The population of this study ( *n* = 103) was composed of men with a mean age of 42.7 (±9.6), with the majority of participants in the 36 to 44 age group (36.9%). The participants were mostly single (55.3%), with 6 to 9 years of schooling (54.4%), and after their arrest, 56 participants were unemployed. Regarding smoking habits, the majority (72.5%) were smokers, with 44.0% of individuals smoking between 11 and 20 cigarettes a day.

Concerning the imprisonment characteristics, the average length of imprisonment for those over 12 and the number of participants who do not receive any visits were also noted.

According to oral dental habits, the majority reported they brushed their teeth (80.6%), and 32 participants did not visit the dentist before the detention, with 20 of these participants not considering it necessary to do so. With regard to oral health, 37.9% of individuals stated that their oral habits were poor, 25.2% fair, and 26.2% good.

In the present study, 68.0% of participants were affected by tooth decay, and the mean DMFT for the sample was 16.88 ± 8.56. The component with the greatest weight was the number of missing teeth, with an average value of 13.00 ± 8.44, followed by the number of decayed teeth, with a mean value of 2.26 ± 3.15, and lastly the number of filled teeth, with 1.99 ± 2.04 of average.

The impact of oral health on quality of life was assessed using the OHIP-14, which had an average score of 13.16 ± 11.79. Of all the OHIP-14 dimensions, psychological discomfort had the highest frequency of inmates who answered “frequently”/“very frequently” (29.6%) and the highest average score (2.57 ± 2.53), followed by physical pain (24.3%) with an average of 2.53 ± 2.16 ( Table 1 ).

**Table 1:** Demographic characteristics of the study population

	*N* (%)
Age (mean ± SD)	42.7 ± 9.6
Age, *n* (%)	
≥ 20 y and ≤ 35 y	29 (28.2)
≥ 36 y and ≤ 44 y	38 (36.9)
≥ 45 y	36 (35.0)
Marital status, *n* (%)	
Single	57 (55.3)
Married	19 (18.4)
Divorced	19 (18.4)
Widowed	8 (7.8)
Education level, *n* (%)	
Did not attend school	4 (3.9)
First cycle a	20 (19.4)
Second cycle b	29 (28.2)
Third cycle c	27 (26.2)
Secondary d	20 (19.4)
College e	3 (2.9)
Professional activity prior to imprisonment, *n* (%)	
Never worked	9 (8.7)
Retired	3 (2.9)
Unemployed	13 (12.6)
Self-employed	19 (18.4)
Employee	59 (57.3)
Professional activity in prison, *n* (%)	
Yes	47 (45.6)
No	56 (54.4)
Criminal history, *n* (%)	
First offense	59 (57.3)
Repeat offense	44 (42.7)
Length of sentence, *n* (%)	
≤ 6 y	12 (11.7)
> 6 y and ≤ 12 y	23 (22.3)
≥ 13 y	68 (66.0)
Visits per month, *n* (%)	
No visits	40 (38.8)
Between 1 and 2 visits per month	32 (31.1)
Between 3 and 4 visits per month	31 (30.1)
Smoking habits, *n* (%)	
Non-smokers	28 (27.2)
Light smokers (≤ 10 sticks)	27 (28.2)
Moderate (11–20 sticks)	33 (32.0)
Heavy (≥ 21 and above)	15 (14.6)
Oral hygiene habits, *n* (%)	
No	11 (10.7)
Yes Sometimes	83 (80.6) 9 (8.7)
Visit to the dentist before detention, *n* (%)	
No	32 (31.1)
Yes	71 (68.9)
Yes, how often? *n* (%)	
Only in case of pain	26 (36.6)
At least once a year	11 (15.5)
Regularly	34 (47.9)
No, why? *n* (%)	
Did not consider it necessary	20 (62.5)
Expensive consultations	5 (15.6)
Did not have time	2 (6.3)
Fear or anxiety	5 (15.6)
After the arrest, visits to the dentist, *n* (%)	
No	4 (3.9
Yes	99 (96.1)
Perception of oral health, *n* (%)	
Poor	39 (37.9)
Fair	37 (35.9)
Good	27 (26.2)

Abbreviations: *n* , number of individuals in the sample; SD, standard deviation.

a 4 years of education.

b 6 years of education.

c 9 years of education.

d 12 years of education.

e Higher education.

When we compare oral health quality of life and the different dimensions with age groups, we find that in terms of functional limitation, physical pain, physical disability, and social disability, those over 44 years of age had higher median values, although these differences were not statistically significant ( Table 2 ).

**Table 2:** Prevalence of the different dimensions of the OHIP-14

OHIP-14 dimensions	Prevalence		Mean ± SD
	Subjects reporting Never/rarely/sometimes *n* (%)	Subjects reporting Often/very often *n* (%)	
Functional limitation	173 (84.0)	33 (16.0)	1.58 ± 2.13
Physical pain	156 (75.7)	50 (24.3)	2.35 ± 2.16
Psychological discomfort	145 (70.4)	61 (29.6)	2.67 ± 2.53
Physical disability	163 (79.1)	43 (20.9)	1.94 ± 2.22
Psychological disability	166 (80.6)	40 (19.4)	2.11 ± 2.30
Social incapacity	195 (94.7)	11 (5.3)	0.86 ± 1.48
Disadvantages	175 (85.0)	31 (15.0)	1.64 ± 2.03
OHIP-14	1173 (81.3)	269 (18.7)	13.16 ± 11.79

Note: Values indicate number of subjects and percentages; OHIP-14, Oral Health Impact Profile-14; mean ± SD mean (standard deviation).

When we compare OHRQoL and the different dimensions with age groups, we find that in terms of functional limitation, physical pain, physical disability and social disability, those over 44 years of age had higher median values, although these differences were not statistically significant ( Table 3 ).

**Table 3:** Comparison of OHRQoL according to age groups (Kruskal–Wallis test)

Dimensions	Age groups (in years)	Median (IQR)	*H*	*p*
Functional limitation	20–35	0.0 [0.0; 2.5]	1.72	0.423
	36–44	0.0 [0.0; 2.25]		
	> 44	1.5 [0.0;3.75]		
Physical pain	20–35	2.0 [0.0;4.0]	0.743	0.690
	36–44	1.5 [1.0;4.25]		
	> 44	3.0 [0.0; 4.0]		
Psychological discomfort	20–35	3.0 [0.0; 5.5]	0.360	0.835
	36–44	3.0 [0.0; 5.25]		
	> 44	2.0 [0.0; 4.0]		
Physical disability	20–35	1.0 [0.0; 3.0]	0.017	0.991
	36–44	2.0 [0.0; 3.0]		
	> 44	0.5 [0.0; 4.0]		
Psychological disability	20–35	2.0 [0.0; 5.0]	1.486	0.476
	36–44	2.0 [0.0; 4.0]		
	> 44	0.5 [0.0; 4.0]		
Social incapacity	20–35	0.0 [0.0; 1.0]	0.334	0.846
	36–44	0.0 [0.0; 2.0]		
	> 44	0.0 [0.0; 2.0]		
Disadvantages	20–35	1.0 [0.0;4.0]	0.609	0.737
	36–44	0.0 [0.0; 3.0]		
	> 44	0.0 [0.0; 3.75]		
OHIP-14	20–35	12.0 [2.0; 23.0]	0.086	0.958
	36–44	11.5 [2.75; 20.0]		
	> 44	9.5 [4.0; 26.00]		

Abbreviations: IQR, interquartile range; *N* , sample number; OHIP-14, Oral Health Impact Profile-14; *U* -statistics and *p* -value derived from Kruskal–Wallis test.

Table 4 shows a comparison between the levels of OHRQoL and different dimensions, according to the smoking habits. The results show that heavy smokers (more than 21 sticks) have a poorer quality of life in all dimensions compared to moderate smokers (between 11 and 20 sticks), light smokers (10 sticks or less). In terms of functional limitation, these differences were statistically significant between none and heavy smokers ( *p* = 0.005) moderate and heavy and moderate smokers ( *p* = 0.011); in terms of physical pain these differences were statistically significant between moderate and heavy smokers ( *p* = 0.027); in psychological discomfort these differences were statistically significant between none and heavy smokers ( *p* = 0.015). With regard to and physical disability, the differences were statistically significant between none and heavy smoker ( *p* = 0.025) and moderate and heavy smokers ( *p* = 0.007). With regard psychological disability, the differences were statistically significant between none and light smokers ( *p* = 0.028), none and heavy smokers ( *p* = 0.004), and between moderate and heavy smokers ( *p* = 0.0041). With regard to social disability, the differences were statistically significant between none and heavy smokers ( *p* = 0.000), between light and heavy smokers ( *p* = 0.029) and between moderate and heavy smokers ( *p* = 0.000). With regard to disadvantage, the differences were statistically significant between none and heavy smokers ( *p* = 0.0019 and between moderate and heavy smokers ( *p* = 0.007), and the same was true of OHIP ( *p* = 0.001 and *p* = 0.004, respectively).

**Table 4:** OHRQoL and smoking habits

	Smoking habits	Median (IQR)	*H*	*p*
Functional limitation	Non-smokers	0.0 [0.0; 2.0] *	12.60	0.006
	Light smokers (≤ 10 sticks)	0.0 [0.0; 3.0]		
	Moderate (11–20 sticks)	0.0 [0.0; 2.0] *		
	Heavy (≥ 21 and above)	3.0 [1.0; 6.0] *		
Physical pain	Non-smokers	1.0 [0.0; 3.75]	10.86	0.012
	Light smokers (≤ 10 sticks)	3.0 [1.0; 4.0]		
	Moderate (11–20 sticks)	1.0 [0.0; 3.0] **		
	Heavy (≥ 21 and above)	3.0 [2.0; 6.0] **		
Psychological Discomfort	Non-smokers	0.0 [0.0; 3.0] ***	11.31	0.010
	Light smokers (≤ 10 sticks)	3.0 [0.0; 6.0]		
	Moderate (11–20 sticks)	2.0 [0.0; 4.0]		
	Heavy (≥ 21 and above)	2.0 [5.0; 6.0] ***		
Physical Disability	Non-smokers	0.0 [0.0; 3.0] ****	12.23	0.007
	Light smokers (≤ 10 sticks)	2.0 [0.0; 4.0]		
	Moderate (11–20 sticks)	0.0 [0.0; 3.0] ****		
	Heavy (≥ 21 and above)	4.0 [1.0; 6.0] ****		
Psychological Disability	Non-smokers	0.0 [0.0; 2.0] *****	15.69	0.001
	Light smokers (≤ 10 sticks)	3.0 [0.0; 5.0] *****		
	Moderate (11–20 sticks)	0.0 [0.0; 2.5] *****		
	Heavy (≥ 21 and above)	4.0 [2.0; 6.0] *****		
Social Incapacity	Non-smokers	0.0 [0.0; 0.0] ******	20.14	< 0.001
	Light smokers (≤ 10 sticks)	0.0 [0.0; 2.0] ******		
	Moderate (11–20 sticks)	0.0 [0.0; 0.0] ******		
	Heavy (≥ 21 and above)	3.0 [0.0; 4.0] ******		
Disadvantages	Non-smokers	0.0 [0.0; 1.75] *******	16.92	< 0.001
	Light smokers (≤ 10 sticks)	2.0 [0.0; 4.0]		
	Moderate (11–20 sticks)	0.0 [0.0; 2.5] *******		
	Heavy (≥ 21 and above)	4.0 [2.0; 5.0] *******		
OHIP-14	Non-smokers	4.5 [1.0; 12.75] ********	18.54	< 0.001
	Light smokers (≤ 10 sticks)	13.0 [5.0; 27.0]		
	Moderate (11–20 sticks)	8.0 [2.5 ;15.5] ********		
	Heavy (≥ 21 and above)	27.0 [17.0; 34.0] ********		

Abbreviations: IQR, interquartile range; *N* , sample number; OHIP-14, Oral Health Impact Profile-14; *H* -statistics and *p* -value derived from Kruskal-Wallis and *p* -value derived from Dunn's test with the Bonferroni correction.

* *p* = 0.005 and *p* = 0.011.

** *p* = 0.027.

*** *p* = 0.015.

**** *p* = 0.025 and *p* = 0.007.

***** *p* = 0.028 and *p* = 0.004 and *p* = 0.0041.

****** *p* = 0.000 and *p* = 0.029 and *p* = 0.000.

******* *p* = 0.0019 and *p* = 0.007.

******** *p* = 0.001 and *p* = 0.004.

According to Table 5 , individuals who do not have oral hygiene habits have the worst OHRQoL in all dimensions, compared with those who brush their teeth sometimes and those who always brush. These differences were statistically significant in functional limitation ( *H* = 8.88; *p* = 0.012) and were established between those who do not brush and those who always brush ( *p* = 0.01); in physical pain ( *H* = 8.48; *p* = 0.014) between those who answered “yes” and those who answered “no” ( *p* = 0.039), in physical disability ( *H* = 8.07; *p* = 0.018) between those who answered “yes” and those who answered “no” ( *p* = 0.049), psychological disability ( *H* = 11.17; *p* = 0.004) between those who answered “yes” and those who answered “no” ( *p* = 0.005), disadvantage ( *H* = 17.26; *p* < 0.001) between those who answered “yes” and those who answered “no” ( *p* = 0.000) and in total OHIP ( *H* = 12.36; *p* = 0.002) between those who answered “yes” and those who answered “no” ( *p* = 0.004).

**Table 5:** OHRQoL and oral hygiene habits

Dimensions	Oral hygiene habits	Median (IQR)	*H*	*p*
Functional limitation	No	4.0 [2.0; 6.0] *	8.88	0.012
	Yes	0.0 [0.0; 2.0] *		
	Sometimes	0.0 [0.0; 4.0]		
Physical pain	No	4.0 [2.0; 6.0] **	8.48	0.014
	Yes	1.0 [0.0; 4.0] **		
	Sometimes	3.0 [2.5; 4.0]		
Psychological discomfort	No	4.0 [0.0; 6.0]	1.97	0.374
	Yes	2.0 [0.0; 4.0]		
	Sometimes	2.0 [1.0; 4.5]		
Physical disability	No	3.0 [2.0; 6.0] ***	8.07	0.018
	Yes	0.0 [0.0; 3.0] ***		
	Sometimes	3.0 [0.0; 5.5]		
Psychological disability	No	5.0 [3.0; 6.0] ****	11.17	0.004
	Yes	1.0 [0.0; 3.0] ****		
	Sometimes	1.0 [1.0; 5.0]		
Social incapacity	No	3.0 [.0; 3.0]	5.92	0.052
	Yes	0.0 [0.0; 1.0]		
	Sometimes	0.0 [0.0; 2.0]		
Disadvantages	No	4.0 [2.0; 6.0] *****	17.26	<0.001
	Yes	0.0 [0.0; 2.0] *****		
	Sometimes	2.0 [1.0; 3.5]		
OHIP-14	No	28.0 [15.0; 34.0] ******	12.36	0.002
	Yes	8.0 [2.0; 18.0] ******		
	Sometimes	15.0 [11.0; 26.5]		

Abbreviations: IQR, interquartile range; *N* , sample number; OHIP-14, Oral Health Impact Profile-14; *H* -statistic and *p* -value derived from Kruskal-Wallis and *p* -value derived from Dunn's test with the Bonferroni correction.

* *p* = 0.01.

** *p* = 0.039.

*** *p* = 0.049.

**** *p* = 0.005.

***** *p* = 0.000.

****** *p* = 0.004.

Individuals with a poor perception of oral health have significantly lower levels of OHRQoL in all dimensions, compared with those with a reasonable and good perception of oral health, assuming statistical significance in all dimensions ( Table 6 ).

**Table 6:** OHRQoL according to perception of oral health

Dimensions	Oral health	Median (IQR)	*H*	*p*
Functional limitation	Poor	3.0 [0.0; 4.0] **	23.93	< 0.001
	Fair	0.0 [0.0; 2.0] **		
	Good	0.0 [0.0; 0.0] **		
Physical pain	Poor	4.0 [3.0; 6.0] ***	30.41	<0.001
	Fair	1.0 [0.0; 3.0] ***		
	Good	1.0 [0.0; 2.0] ***		
Psychological discomfort	Poor	4.0 [2.0; 6.0] ****	15.98	<0.001
	Fair	2.0 [0.0; 4.0] ****		
	Good	0.0 [0.0; 3.0] ****		
Physical disability	Poor	3.0 [2.0; 6.0] *****	25.54	< 0.001
	Fair	0.0 [0.0; 2.0] *****		
	Good	0.0 [0.0; 2.0] *****		
Psychological disability	Poor	3.0 [0.0; 5.0] ******	14.39	< 0.001
	Fair	1.0 [0.0; 3.0] ******		
	Good	0.0 [0.0; 2.0] ******		
Social incapacity	Poor	2.0 [0.0; 3.0] *******	17.40	< 0.001
	Fair	0.0 [0.0; 0.0] *******		
	Good	0.0 [0.0; 0.0] *******		
Disadvantages	Poor	3.0 [1.0; 4.0] ********	26.02	< 0.001
	Fair	0.0 [0.0; 2.0] ********		
	Good	0.0 [0.0; 2.0] ********		
OHIP-14	Poor	23.0 [12.0; 28.0] *	34.43	< 0.001
	Fair	6.0 [2.5; 13.0] *		
	Good	3.0 [1.0; 9.0] *		

Abbreviations: IQR, interquartile range; *N* , sample number; OHIP-14, Oral Health Impact Profile-14; *H* -statistic and *p* -value derived from Kruskal-Wallis and *p* -value derived from Dunn's test with the Bonferroni correction.

* Statistically significant differences in OHIP-14 between fair and poor ( *p* = 0.000) and between good and poor ( *p* = 0.000).

** Statistically significant differences in functional limitation between fair and poor ( *p* = 0.000) and good and poor ( *p* = 0.000).

*** Statistically significant differences in physical pain between fair and poor ( *p* = 0.000) and between good and poor ( *p* = 0.000).

**** Statistically significant differences in psychological discomfort between reasonable and poor ( *p* = 0.001) and good and poor ( *p* = 0.01).

***** Statistically significant differences in physical disability between reasonable and poor ( *p* = 0.000) and between good and poor ( *p* = 0.000).

****** Statistically significant differences in psychological disability between reasonable and poor ( *p* = 0.001) and good and poor ( *p* = 0.02).

******* Statistically significant differences in social disability between reasonable and poor ( *p* = 0.000) and good and poor ( *p* = 0.008).

******** Statistically significant differences in disadvantage between reasonable and poor ( *p* = 0.000) and between good and poor ( *p* = 0.000).

When comparing quality of life with oral health according to caries lesion ( Table 7 ), it was found that individuals with caries lesions have a worse quality of life with oral health in all dimension compared with those without caries lesions, with these differences being statistically significant in psychological discomfort ( *U* = 570.5; *p* = 0.004), physical disability ( *U* = 661.0; *p* = 0.03), psychological disability ( *U* = 598.0; *p* = 0.007), social disability ( *U* = 688.0; *p* = 0.027) a total OHIP-14 ( *U* = 576.5; *p* = 0.006).

**Table 7:** Comparison of quality of life with oral health according to caries lesion

Dimensions	Caries lesions	*N*	Median (IQR)	*U*	*p*
Functional limitation	No	26	0.0 [0.0; 2.0]	713.0	0.072
	Yes	70	0.0 [0.0; 3.0]		
Physical pain	No	26	2.0 [0.0; 3.25]	806.5	0.384
	Yes	70	2.0 [0.0; 4.0]		
Psychological discomfort	No	26	0.5 [0.0; 3.0]	570.5	0.004
	Yes	70	3.0 [0.0; 6.0]		
Physical disability	No	26	0.0 [0.0; 2.0]	661.0	0.03
	Yes	70	2.0 [0.0; 4.0]		
Psychological disability	No	26	0.0 [0.0; 2.0]	598.0	0.007
	Yes	70	2.0 [0.0; 4.25]		
Social incapacity	No	26	0.0 [0.0; 0.0]	688.0	0.027
	Yes	70	0.0 [0.0; 3.25]		
Disadvantages	No	26	0.0 [0.0; 2.0]	707.0	0.067
	Yes	70	1.0 [0.0; 4.0]		
OHIP-14	No	26	4.0 [1.0; 12.25]	576.5	0.006
	Yes	70	12.0 [4.0; 24.25]		

Abbreviations: IQR, interquartile range; *N* , sample number; OHIP-14, Oral Health Impact Profile-14; *U* -statistics and *p* -value derived from Mann–Whitney test.

In assessing quality of life related to periodontal disease, individuals with gingivitis showed the poorest outcomes in functional limitation and physical pain, though differences were not statistically significant compared with those with periodontitis and periodontal health. However, significant differences were observed in psychological discomfort ( *H* = 8.00; *p* = 0.018) between individuals with periodontitis and those with periodontal health, and between gingivitis and periodontitis ( *p* = 0.025); psychological disability ( *H* = 6.49; *p* = 0.039) between periodontal health and periodontitis ( *p* = 0.049); and total OHIP ( *H* = 7.81; *p* = 0.02) between periodontal health and periodontitis ( *p* = 0.018). Individuals with periodontitis generally exhibited the poorest quality of life across domains ( Table 8 ).

**Table 8:** Comparison of quality of life with periodontal health and its category

Dimensions	Periodontal health	*N*	Median (IQR)	*H*	*p*
Functional limitation	Healthy periodontium	24	0.0 [0.0; 1.5]	5.00	0.082
	Gingivitis	22	1.0 [0.0; 3.0]		
	Periodontitis	50	0.5 [0.0; 3.0]		
Physical pain	Healthy periodontium	24	0.5 [0.0; 3.75]	2.59	0.274
	Gingivitis	22	2.0 [1.0; 4.0]		
	Periodontitis	50	2.0 [0.0; 4.0]		
Psychological discomfort	Healthy periodontium	24	1.5 [0.0; 3.0]	8.00	0.018
	Gingivitis	22	0.5 [0.0; 3.25] *		
	Periodontitis	50	3.0 [0.75; 6.0] *		
Physical disability	Healthy periodontium	24	0.0 [0.0; 2.0]	3.98	0.137
	Gingivitis	22	1.0 [0.0; 3.0]		
	Periodontitis	50	2.0 [0.0; 4.0]		
Psychological disability	Healthy periodontium	24	0.5 [0.0; 2.0] **	6.49	0.039
	Gingivitis	22	0.0 [0.0; 4.25]		
	Periodontitis	50	2.0 [0.0; 4.0] **		
Social incapacity	Healthy periodontium	24	0.0 [0.0; 0.0]	2.83	0.243
	Gingivitis	22	0.0 [0.0; 1.0]		
	Periodontitis	50	0.0 [0.0; 2.0]		
Disadvantages	Healthy periodontium	24	0.0 [0.0; 1.0]	4.96	0.084
	Gingivitis	22	0.0 [0.0; 3.25]		
	Periodontitis	50	2.0 [0.0; 3.25]		
OHIP-14	Healthy periodontium	24	5.5 [1.0; 12.0] ***	7.81	0.02
	Gingivitis	22	9.5 [2.0; 22.5]		
	Periodontitis	50	14.0 [4.0; 24.25] ***		

Abbreviations: IQR, interquartile range; *N* , sample number; OHIP-14, Oral Health Impact Profile-14; *H* -statistics and *p* -value derived from Kruskal-Wallis and *p* -value derived from Dunn's test with the Bonferroni correction

* Statistically significant differences in psychological distress between individuals with gingivitis and periodontitis ( *p* = 0.025).

** Statistically significant differences in psychological disability between individuals with periodontal health and periodontitis ( *p* = 0.049)

*** Statistically significant differences in OHIP Total between individuals with periodontal health and periodontitis ( *p* = 0.018).

The relationship between the OHIP-14 dimensions and the periodontal indices and the number of missing teeth is demonstrated in Table 9 . The DMFT index was found to have a weak and statistically significant positive correlation with all the dimensions and with total OHIP-14. The PI showed a weak positive correlation with all dimensions, but this relationship was only statistically significant with social disability ( *r* = 0.201; *p* < 0.05) and disadvantage ( *r* = 0.214; *p* < 0.05). The BOP was weakly correlated, showing statistically significant differences with psychological discomfort ( *r* = 0.273; *p* < 0.01), psychological disability ( *r* = 0.226; *p* < 0.05), social disability ( *r* = 0.246; *p* < 0.05 and total OHIP-14 ( *r* = 0.263; *p* < 0.01). Mean PD correlated weakly and statistically significantly with psychological discomfort ( *r* = 0.299; *p* < 0.01), psychological disability ( *r* = 0.303; *p* < 0.01), social disability ( *r* = 0.293; *p* < 0.01), and total OHIP-14 (r = 0.287; *p* < 0.01). Mean CAL correlated weakly and statistically significantly with all dimensions except physical disability. The number of missing teeth also correlated weakly and statistically significantly with all dimensions. In conclusion, we can see that the higher the value of the different periodontal indices, as well as the number of missing teeth, the worse the quality of life in terms of oral health.

**Table 9:** Relationship between OHIP-14 dimensions and periodontal indices and number of missing teeth (Spearman correlation, *n* = 103)

Dimensions	DMFT Index	PI	BOP	PD Average	CAL Average	No. of teeth lost
Functional limitation	0.434 a	0.047	0.191	0.173	0.280 a	0.442 a
Physical pain	0.303 a	0.071	0.162	0.167	0.239 b	0.305 a
Psychological discomfort	0.231 b	0.210 b	0.273 a	0.299 a	0.290 a	0.243 b
Physical disability	0.319 a	0.029	0.178	0.166	0.162	0.314 a
Psychological disability	0.313 a	0.183	0.226 b	0.303 a	0.289 a	0.307 a
Social incapacity	0.296 a	0.201 b	0.246 b	0.293 a	0.345 a	0.223 b
Disadvantages	0.445 a	0.214 b	0.183	0.198	0.207 b	0.415 a
OHIP-14	0.418 a	0.169	0.263 a	0.287 a	0.323 a	0.406 a

Abbreviations: BOP, bleeding on probing; CAL, clinical attachment loss; DMFT Index, Decayed, Missing, and Filled Permanent Teeth Index; OHIP-14, Oral Health Impact Profile-14; PD, probing depth; PI, Plaque Index; .

a *p* < 0.05.

b *p* < 0.01.

## Discussion

The existence of problems associated with oral health has been shown to be one of the main factors causing a negatively impact daily activities. This leads to reduced social interaction, physical and psychological problems and, consequently, pain and suffering. As the prison population is deprived of their liberty, the problems are exacerbated and can have a negative impact on the quality of life. To develop new scientific data and given the scarcity of studies on oral health-related quality of life in the prison population in Portugal, this study was fundamental to ascertaining the need to create measures to promote oral health.

The study sample consisted of a total of 103 individuals from a prison in northern Portugal. In agreement with the studies conducted by Reddy et al, 12 Fotedar et al, 13 and Nobile et al 20 ; the average age of the sample was 42.7 years, with the majority in the age group of 36 to 44 years.

To access the impact of oral health on quality of life, the OHIP-14 questionnaire was used, as in studies conducted in India, 13 Scotland, 21 and Brazil, 4 where the mean values for impact on OHRQoL ranged from 14.57 to 19.16, higher than in our sample. Of all the OHIP-14 dimensions, the predominance of the psychological discomfort dimension in relation to the prevalence of greater impact is compatible with the results of Freeman et al, 21 who found an average value of 3.20. Physical pain was the second dimension with a significant impact, which is in line with the high percentage of inmates who reported experiencing a toothache. Studies by Moraes et al 14 and Fotedar et al 13 also found a high percentage of prevalence in the physical pain dimension, with values of 40.4 and 21.8%, respectively.

The age factor has a major influence on quality of life. As older individuals are more likely to develop health problems, they are at greater risk of losing tooth structure, affecting chewing, speech, or social integration. In terms of OHIP prevalence, Soares et al 4 observed that individuals between 45 and 54 years of age have a greater impact on OHRQoL, showing lower levels of quality of life. As in our study, those aged over 44 had a bigger impact in terms of functional limitation, physical pain, physical disability, and social disability, although the differences were not statistically significant.

Tobacco consumption is the main cause of various oral health issues, which have a significant impact on quality of life, showing that participants who do not have smoking habits have better OHRQoL in all dimensions. These results are similar to those of McGrath C, 22 in which 94.0% of inmates who smoked had a worse quality of life. The prevalence has been increasing worldwide, and studies conducted in London, 23 Italy, 20 India, 12 and Brazil 14 have demonstrated this significant increase. Relating to the prison population included in the study, there was a high percentage of smokers, with the majority smoking between 11 and 20 cigarettes a day. The study by Huilgol et al 24 showed a strong and consistent link between the number of cigarettes smoked per day and deterioration in oral health. This relationship is evident in the increased risks of periodontal disease, tooth loss, and oral cancer, which have a significant impact on quality of life.

When assessing oral health in a London prison, Heidari et al 23 found that the majority of participants mentioned that their oral health was poor and that they needed treatment. The study by Moraes et al, 14 which assessed self-related health and its effects on quality of life, revealed that those who rated their health as poor had a higher average score for impact on quality of life. These findings are consistent with our results, in which OHRQoL levels were significantly lower in individuals who had a poor perception of oral health, showing statistical significance in all dimensions, compared with those who had a reasonable and good perception of oral health.

The prevalence of oral diseases in the prison was significantly high. As in other studies, 1 4 11 12 13 dental caries and periodontitis were the most prevalent oral diseases. Fotedar et al 13 emphasized the need for proper oral care education to reduce the high prevalence the oral diseases, as did Reddy et al, 12 who found that 97.7% of the participants needed oral hygiene instructions.

Periodontitis statistically interfered with OHRQoL, with statistical significance in the dimensions of psychological discomfort, psychological disability, and OHIP-14 total, compared with the groups with gingivitis and periodontal health. The presence of an impact on quality of life was reported more frequently by participants in the study by Soares et al, 4 who had severe periodontitis.

The study by Rouxel et al 11 made it possible to access the oral conditions of the prison population in comparison to the general population. According to their results, the inmates had a high percentage of caries lesions, and 73% of the participants had at least one oral impact, showing difficulties with chewing, relaxation, emotional stability, and social interaction. These results validate the fact that individuals with caries lesions have worse levels of quality of life, with statistically significant differences in psychological discomfort, physical disability, psychological disability, and total OHIP-14 scores.

This study had some limitations that should be considered for potential future research. One of the limitations was the small sample and the specific geographical area which can be considered unrepresentative of the entire inmate population in Portugal. In relation to the questionnaires conducted, it should be considered that these results are based on the responses provided by participants, which is another limitation of the study since the responses may not always correspond to reality.

This study significantly contributes to the understanding of oral health-related quality of life (OHRQoL) among the prison population in Portugal, highlighting the critical need for targeted oral health interventions within correctional facilities. The results highlight the high prevalence of oral diseases, such as dental caries and periodontitis, negative impact on various dimensions of quality of life, particularly psychological discomfort and physical pain. Clinically, these results support implementing comprehensive oral health education programs and preventive measures suited to prisoners, focusing on the importance of addressing tobacco use, which is strongly associated with negative oral health outcomes. By identifying the specific oral health challenges faced by detained individuals, this study provides a framework for health professionals and policymakers to develop effective strategies aimed at improving oral health services, enhancing the overall well-being of inmates, and potentially facilitating their reintegration into society.

## Conclusion

The impact of quality of life in relation to oral health in the EPPF prison population showed an average value of 13.16 ± 11.79, with psychological discomfort being the dimension with the greatest impact, followed by physical pain. The prevalence of periodontal diseases, such as periodontitis, and the high percentage of inmates with caries lesions are two oral problems that negatively affect the quality of life in prisons. Most of the participants had a poor self-perceived oral health, emphasizing a great concern for the creation of preventive and educational measures as a way of reducing risk factors that could develop oral health problems.

## References

[1] Longhi M TM Silva R UO Gasque K CDS Lima D C Oliveira J M Caldeira F ID Impact of oral comorbidities on incarcerated women: an integrative review Rev Esp Sanid Penit 2022 24 03 94 100 10.18176/resp.00057 PMC9768559 36533787

[2] World Health Organization. The World Health Organization Quality of Life (WHOQOL) (2012). Accessed October 16, 2025 at:https://www.who.int/publications/i/item/WHO-HIS-HIS-Rev.2012.03

[3] Khademi N Zanganeh A Saeidi S Quality of life of HIV-infected individuals: insights from a study of patients in Kermanshah, Iran BMC Infect Dis 2021 21 01 203 10.1186/s12879-021-05908-z PMC7903600 33622262

[4] Soares G H Mendonça I Michel-Crosato E Moysés S J Moysés S T Werneck R I Impact of oral conditions on the quality of life of incarcerated women in Brazil Health Care Women Int 2019 40(7-9):776 787 10.1080/07399332.2019.1590362 30901282

[5] Thetakala R K Chandrashekar B R Sunitha S Maurya M Sharma P Shubhi G Bruxism and oral health-related quality of life among male inmates in a penal institution, Mysore: a cross-sectional study Indian J Dent Res 2018 29 03 275 279 10.4103/ijdr.IJDR_203_17 29900908

[6] Marshman Z Baker S R Robinson P G Does dental indifference influence the oral health-related quality of life of prisoners? Community Dent Oral Epidemiol 2014 42 05 470 480 10.1111/cdoe.12097 24450539

[7] Orjiakor C T Ugwu D I Eze J E Ugwu L I Ibeagha P N Onu D U Prolonged incarceration and prisoners' wellbeing: livid experiences of awaiting trial/pre-trial/remand prisoners in Nigeria Int J Qual Stud Health Well-being 2017 12 01 1.395677E6 10.1080/17482631.2017.1395677 PMC5678456 29103371

[8] Bellali T Manomenidis G Karamitri I Malliarou M Igoumenou A Bellali N Health-related quality of life of prisoners with diabetes mellitus J Forensic Leg Med 2020 73 101971 10.1016/j.jflm.2020.101971 32658745

[9] Soares M Gonçalves M Rompante P Assessment of oral health status in a prison population in Northern Portugal J Clin Exp Dent 2023 15 11 e912 e919 10.4317/jced.60551 PMC10699763 38074165

[10] Relvas M Rocha L Rompante P Oral lesions and disorders and their prevalence arising from the use of illicit drugs in a prison population Acta Odontol Scand 2025 84 37 46 10.2340/aos.v84.42721 PMC11808817 39834209

[11] Rouxel P Duijster D Tsakos G Watt R G Oral health of female prisoners in HMP Holloway: implications for oral health promotion in UK prisons Br Dent J 2013 214 12 627 632 10.1038/sj.bdj.2013.581 23787853

[12] Reddy V Kondareddy C V Siddanna S Manjunath M A survey on oral health status and treatment needs of life-imprisoned inmates in central jails of Karnataka, India Int Dent J 2012 62 01 27 32 10.1111/j.1875-595X.2011.00082.x PMC9374940 22251034

[13] Fotedar S Chauhan A Bhardwaj V Manchanda K Fotedar V Association between oral health status and oral health-related quality of life among the prison inmate population of Kanda Model Jail, Shimla, Himachal Pradesh, India Indian J Public Health 2016 60 02 150 153 10.4103/0019-557X.184573 27350711

[14] Moraes L R Duarte de Aquino L C da Cruz D T Leite I CG Self-perceived impact of oral health on the quality of life of women deprived of their liberty Int J Dent 2021 2021 5.520652E6 10.1155/2021/5520652 PMC8178007 34135966

[15] Cuschieri S The STROBE guidelines Saudi J Anaesth 2019 13 01 S31 S34 10.4103/sja.SJA_543_18 PMC6398292 30930717

[16] Afonso A Silva I Meneses R Frias-Bulhosa J Oral health-related quality of life: Portuguese linguistic and cultural adaptation of OHIP-14 [in Portuguese] Psicol Saude Doencas 2017 18 02 374 388

[17] Tonetti M S Greenwell H Kornman K S Staging and grading of periodontitis: Framework and proposal of a new classification and case definition J Periodontol 2018 89 01 S159 S172 10.1002/JPER.18-0006 29926952

[18] Ainamo J Bay I Problems and proposals for recording gingivitis and plaque Int Dent J 1975 25 04 229 235 1058834

[19] O'Leary T J Drake R B Naylor J E The plaque control record J Periodontol 1972 43 01 38 10.1902/jop.1972.43.1.38 4500182

[20] Nobile C GA Flotta D Nicotera G Pileggi C Angelillo I F Self-reported health status and access to health services in a sample of prisoners in Italy BMC Public Health 2011 11 529 10.1186/1471-2458-11-529 PMC3151234 21726446

[21] Freeman R Richards D Factors associated with accessing prison dental services in Scotland: a cross-sectional study Dent J 2019 7 01 12 10.3390/dj7010012 PMC6473453 30717283

[22] McGrath C Oral health behind bars: a study of oral disease and its impact on the life quality of an older prison population Gerodontology 2002 19 02 109 114 10.1111/j.1741-2358.2002.00109.x 12542220

[23] Heidari E Dickinson C Wilson R Fiske J Verifiable CPD paper: oral health of remand prisoners in HMP Brixton, London Br Dent J 2007 202 02 E1 10.1038/bdj.2007.32 17235361

[24] Huilgol P Bhatt S P Biligowda N Wright N C Wells J M Association of e-cigarette use with oral health: a population-based cross-sectional questionnaire study J Public Health (Oxf) 2019 41 02 354 361 10.1093/pubmed/fdy082 PMC6636695 29788415

